# The course of depressive symptoms with decline in cognitive function - a longitudinal study of older adults receiving in-home care at baseline

**DOI:** 10.1186/s12877-019-1226-8

**Published:** 2019-08-23

**Authors:** Anne-Sofie Helvik, Maria Lage Barca, Sverre Bergh, Jūratė Šaltytė-Benth, Øyvind Kirkevold, Tom Borza

**Affiliations:** 1 General Practice Research Unit, Department of Public Health and Nursing, Faculty of Medicine and Health Sciences, Norwegian University of Science and Technology (NTNU), Postboks 8905, NO-7491 Trondheim, Norway; 2 Norwegian National Advisory Unit on Ageing and Health, Vestfold Hospital Trust, Tønsberg, Norway; 3 St Olavs University Hospital, Trondheim, Norway; 4 Research Centre for Age-related Functional Decline and Disease, Innlandet Hospital Trust, Ottestad, Norway; 5 Institute of Clinical Medicine, University of Oslo, Campus Ahus, Oslo, Norway; 6 Health Services Research Unit, Akershus University Hospital, Lørenskog, Norway; 7 Department of Health Sciences, Faculty of Medicine and Health Sciences, Norwegian University of Science and Technology (NTNU), Gjøvik, Norway

**Keywords:** Cognitive decline, CSDD, Depressive symptoms, Depression, Domiciliary care, Elderly, Home care, Neuropsychiatric inventory, Symptom load, Physical disease

## Abstract

**Background:**

Depressive symptoms in old age are common, but the prevalence, persistence, and incidence of depressive symptoms in older adults with and without dementia receiving in-home care is less well studied, and descriptions of the relationship between severity of cognitive decline and depressive symptoms over time is, to our knowledge, lacking. The aim of the present study was to describe the prevalence, incidence and persistence of depressive symptoms over a 36-month follow-up period among older adults receiving in-home care at baseline, and to explore the association between cognitive function and the course of depressive symptoms over time.

**Methods:**

In all, 1001 older people (≥ 70 years) receiving in-home care were included in a longitudinal study with three assessments over 36 months. Depressive symptoms were assessed using the Cornell Scale for Depression in Dementia. Clinical Dementia Rating Scale, diagnosis of dementia and mild cognitive impairment, general medical health, personal and instrumental activities of daily living, neuropsychiatric symptoms and the use of psychotropic medication were evaluated during the three assessments. Baseline demographic characteristics and information on nursing home residency at follow-up were recorded. Linear mixed models were estimated.

**Results:**

The baseline prevalence and cumulative incidence of single depressive symptoms were higher in those with dementia at baseline than in those without dementia. The persistence of depressive symptoms did not differ between those with or without dementia at baseline. The severity of cognitive impairment and mean depressive symptom score assessed simultaneously were positively associated, but the strength of the association changed over time and was not significant at the last assessment. Furthermore, being younger, female, in very poor physical health, with neuropsychiatric symptoms and not becoming a nursing home resident were associated with more depressive symptoms when assessed simultaneously.

**Conclusion:**

The baseline prevalence and cumulative incidence of depressive symptoms in those with and without dementia at baseline, as well as the relationship we found between the degree of cognitive decline and depressive symptoms over time show that depression and dementia are interconnected. Nurses and clinicians should pay attention to cognitive status when observing or evaluating depression among older adults receiving in-home care.

## Introduction

Depression is a frequent cause of emotional suffering in old age, and it may also contribute to reduced physical functioning, reduced quality of life [1–3], increased risk of nursing home admission [4], and reduced life expectancy [5, 6]. Depression in old age is also related to higher health care costs [7].

A population-based meta-analysis found the diagnostic pooled prevalence of depression in older adults to be about 7% [8]. A review of population-based studies of older adults with mild cognitive impairment (MCI) reported the prevalence of diagnosed depression in these studies to vary between 11 and 40% [9]. In a European study that included data from older adults with dementia in eight countries who were receiving in-home care, the pooled prevalence of clinically significant depressive symptoms, defined as ≥10 on the Cornell Scale for Depression in Dementia, was 34% [10]. The prevalence of clinically significant depressive symptoms in older adults with dementia receiving in-home care varied from 11 to 60% between countries [10, 11]. In some countries the reported prevalence was higher in individuals receiving in-home care than in nursing home (NH) residents [10], but not in all [10, 11]. The prevalence of depression and clinically significant depressive symptoms, as well as the persistence, incidence and remission of depression and depressive symptoms has been extensively explored in NH residents [12, 13], compared to older adults who live at home and receive care. To our knowledge, there are no reports of studies that provide a detailed description of the prevalence, persistence, incidence and remission of individual depressive symptoms in older adults receiving in-home care in the Nordic countries or Norway. However, this information is important for politicians and health care planners in these countries as they plan for providing adequate care for older adults in need of in-home care. The Cornell Scale for Depression in Dementia (CSDD) [14], which is validated for use in people with and without dementia [14–17] and is widely used in clinical settings and research [18–20], is a useful tool to assess depressive symptoms among older adults receiving in-home care.

A review of studies of the course of depression in community dwelling and general practice people has reported that one of three people develop a chronic depressive course [21]. The same review found that factors associated with persistent depression in older community-dwelling residents were greater age, high external locus of control and baseline physical co-morbidity, functional limitations, and more severe depressive symptoms, but the evidence was inconclusive when it came to an association between dementia and persistent depression [21]. However, dementia and depression commonly coexist [22], and both a history of depression and use of antidepressants have in reviews been found linked to increased risk for dementia [23, 24]. Furthermore, depressive symptoms may present somewhat differently in people with dementia compared to people without dementia [25]. Not all people with MCI develop dementia, but a review of studies exploring depression in older adults with MCI concluded that MCI could be the earliest identifiable clinical stage of dementia, and argued that the underlying neuropathological condition causing MCI or dementia may also cause depressive symptoms [9]. It is possible that late-life depression could be an early cause prodromal sign of dementia, especially because pathological changes associated with dementing diseases can begin long before their clinical onset [6]. Thus, the course of depressive symptoms in older adults receiving in-home care is of particular interest and may give us a better understanding of the relationship between cognitive decline, and the course of depressive symptoms. In clinical practice, dementia may be mistaken for depression due to lack of initiative and limitations in personal and instrumental activities of daily living.

To explore the course of depressive symptoms, we have carried out a longitudinal, large-scale study of a group of older adults receiving in-home care at baseline, with three assessments over 36 months. The first aim of our study was to describe the prevalence, incidence and persistence of depressive symptoms over the follow-up period, and the second aim was to explore the association between cognitive function and the course of depressive symptoms. A detailed description of the prevalence, persistence, incidence and remission of individual depressive symptoms in older adults receiving in-home care is of clinical importance for nurses and other caregivers providing the in-home care. Furthermore, it is of interest to explore the relationship between cognitive functioning and depressive symptoms to improve treatment and follow-up. Politicians and health care planners will favour such knowledge to provide sufficient care for older adults in need of in-home care.

## Methods

### Study design

This longitudinal study, with a 36-month follow-up period, collected baseline information between August 2008 and December 2010. The follow-up assessments were after 18 and 36 months, last follow-up January 2014 [26].

### Participants

In total, 1796 individuals (age ≥ 70 years) from 19 small, medium and large municipalities in five counties in the eastern part of Norway were invited to participate in the study. All participants had to be receiving in-home care, aged ≥70 years and have a next of kin who saw them at least once a week. No further exclusion criteria were included. Participants were both established and new users of in-home care. Established users were drawn from the lists of care service providers and new users were included successively. These services could typically include ‘meals on wheels’, safety alarm, practical aid, day-care centre, mental health care or in-home nursing. The individuals were invited regardless of amount and kind of service received.

Of the total 1796 people invited, 795 declined to participate, leaving 1001 people in the study. Those who declined participation were more often female and older than those included in the study [26, 27].

### Measures

*Depressive symptoms* (dependent variable) were assessed with the Cornell Scale for Depression in Dementia (CSDD) at all assessments [14]. The CSDD was scored by a research assistant after an interview with the patient’s primary caregiver. The CSDD consists of 19 items, with each item rated as 0 (absent), 1 (mild), 2 (severe) or “symptom is not possible to evaluate”. If more than 20% of the items in CSDD were scored as “not possible to evaluate”, the participant was excluded from the analysis, otherwise the item response was set to missing. Since symptoms that are not possible to evaluate may contribute to reducing the sum-score, a mean-score of the scale was used. A clinically significant symptom level indicating depression was defined as a mean CSDD score higher than 0.42, which is equivalent to a sum-score cut-off of 9 and higher for CSDD.

*The levels of personal and instrumental functioning* were reported by next of kin and classified using Lawton and Brody’s Physical Self-Maintenance Scale (P-ADL) and Instrumental Activities of Daily Living Scale (I-ADL). The P-ADL sum-score is based on six items (range 6–30), where higher scores indicate a lower level of functioning, while the I-ADL sum-score is based on eight items (range 0–8), where a higher score indicates better I-ADL functioning [28]. These scales are among the shorter scales that are recommended to assess P-ADL and I-ADL [29], are frequently used in Norwegian and Scandinavian studies [30, 31] and are suitable for use in community-living older persons as well as in NH residents [32, 33].

*Cognitive function and dementia severity symptoms* were evaluated by the following tools: Mini-Mental State Examination (MMSE) [34], Clock-Drawing Test (CDT) [35], the Informant Questionnaire on Cognitive Decline in Elderly (IQCODE) [36] and Clinical Dementia Rating Scale (CDR). The CDR includes six domains; memory, orientation, judgement and problem solving, community affairs, home functions, and personal care. An algorithm gives a total score of 0, 0.5, 1, 2 or 3, indicating respectively; no dementia, possible dementia, and mild, moderate and severe dementia [37]. In the present study, we used CDR sum of boxes (CDR-SoB), with a score ranging from 0 to 18, where a higher score indicates more severe cognitive decline [38]. The assessment tools for cognitive function and severity of dementia have been translated and validated in Norwegian [39–41]. The research assessors completed the CDR scale based on all collected information.

*Neuropsychiatric symptoms* were evaluated using the Neuropsychiatric Inventory (10-item NPI) [42] in a translated and validated Norwegian version [43]. The 10-item version covers the following symptoms: delusion, hallucination, euphoria, agitation/aggression, disinhibition, irritability/lability, depression/dysphoria, anxiety, apathy/indifference, and aberrant motor behaviour. Three sub-syndromes of NPI; “Agitation”, “Psychosis”, and “Affective symptoms”, have previously been reported using the sample in the present study [44]. “Agitation” includes the items agitation/aggression, euphoria, disinhibition, aberrant motor behaviour and irritability; “psychosis” includes the items delusions and hallucinations. “Affective symptoms” includes the items depression, anxiety, and apathy.

*Dementia and mild cognitive impairment (MCI)* were diagnosed independently by two experienced physicians in geriatric psychiatry at all three assessments based on all available information. Diagnoses of dementia were made according to the ICD-10 criteria [45] and MCI according to the Winblad criteria [46]. In cases of disagreement, consensus was reached after consulting a third clinical expert.

*Physical health* was evaluated using the General Medical Health Rating Scale (GMHR), considering the patient’s number and severity of medical conditions and the use of medication due to these conditions. GMHR is scored from 1 to 4, where 1 indicates very poor physical health and 4 indicates good physical health [47].

*Data on the participants’ use of drugs* were collected from medical records. Drugs were divided into groups according to the Anatomical Therapeutic Chemical (ATC) Classification System [48]. Psychotropic drugs were categorized as antipsychotics (N05A except lithium), anxiolytics (N05B), hypnotics/sedatives (N05C), antidepressants (N06A), and anti-dementia medication (N06D). Use of medication was dichotomized into yes or no. Medication dosages were not available for the present study.

*The formal level of care* at follow-up assessments was recorded as location, i.e. community-dwelling receiving in-home care or NH care.

*Demographic data* including age, gender, municipality of residence and marital status were recorded in the baseline assessment.

### Procedure

The process of collecting data was led by a research nurse who cooperated with assessors in the different municipalities. The majority of the assessors were nurses, social educators and occupational therapists. All assessors took a two-day training course on how to use the assessment scales before the baseline data collection began. A one-day training course was conducted prior to the second and third assessments. The participants and their next of kin were interviewed simultaneously by two separate research assessors. Further information regarding the data collection is published elsewhere [27].

Study information, both oral and written, was given to the participants and their next of kin. Written informed consent was required from both the participant and their next of kin before the interviews were carried out. In those lacking capacity to consent, their closest family proxy gave their informed consent on behalf of their next of kin. The Regional Committee for Medical and Health Research Ethics for Eastern Norway (S-08111b), the Norwegian Social Science Data Services (NSD) (07–2008SI), and the Directorate for Health and Social Affairs (08/2984) approved this study.

### Data analysis

Sample characteristics at baseline were presented as means and standard deviations (SD) or as frequencies and percentages. For analyses on the course of depressive symptoms, the 19 CSDD items were dichotomized to present (rating of 1 or 2) or absent (rating of 0). Prevalence, incidence, cumulative incidence and persistence were calculated for each depressive symptom and compared between participants with dementia and participants without dementia with a generalized linear mixed-model adjusted for within-municipality correlations. Prevalence was defined as the proportion of residents with the symptom present at an individual assessment. Incidence rate was defined as the ratio of residents who presented the symptom at one assessment to those who were symptom free at the preceding assessment. Cumulative incidence was reported as incidence throughout the whole study period. Persistence rate was calculated as the ratio of residents who presented the symptom at one assessment to those who presented the symptom at the preceding assessment.

To assess if the severity of the cognitive impairment/dementia (CDR-SoB) was associated with mean depressive symptoms (CSDD) over time, two linear mixed models were estimated. Model 1 for CSDD measured at baseline contained fixed effects for baseline CDR-SoB, while CDR-SoB measured simultaneously with longitudinally assessed CSDD was included as a fixed effect in Model 2. Bivariate models with time, CDR-SoB and interaction between those two were estimated first. Next, the models were adjusted for pre-defined covariates (gender, age, single, GMHR, P-ADL, I-ADL, Neuropsychiatric sub-syndrome score for Agitation, Psychosis and Affective, use of antipsychotics, antidepressants, anxiolytics, sedatives, and cognitive enhancers in both models; admission to NH only in Model 2) one at the time. Covariates measured at baseline were included in Model 1, while covariates measured simultaneously with CSDD whenever possible were included in Model 2. In this way, Model 2 takes into account the dynamics in both outcome and covariates. Finally, multiple models that included all covariates were estimated and reduced by applying Akaike’s Information Criterion (AIC), where a smaller value means a better model. All models contained random intercepts for participants nested within municipality and random slope for time. Prior to regression analyses, correlations among assessed variables were evaluated to identify possible multicollinearity issues.

Statistical analyses were performed in SAS v 9.4 and SPSS v 25. Results with *p*-values below 0.05 were considered statistically significant.

## Results

### Sample characteristics

**Fig. 1:**
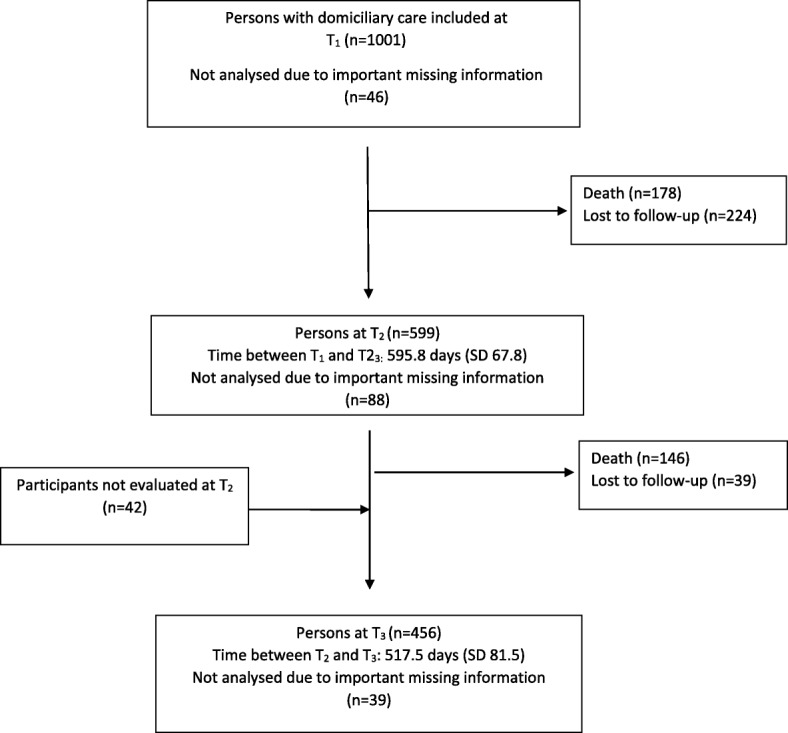
Flowchart for participants over 36 months

Of the 1001 participants at baseline (T_1_), 46 (4.5%) were excluded due to missing data for CSDD (20% or more of single items were not possible to evaluate) at baseline. Among the remaining 955 participants, 394 (41.2%) had dementia, 266 (27.9%) had MCI and 295 (30.9%) had no cognitive impairment at baseline, and the mean (SD) age was 83.4 (5.6) years (see Table 1). In all, 653 (68.4%) of the participants were women, 287 (30.1%) were married and 149 (15.6%) had a good physical health based on the GMHR. The mean (SD) baseline CDR-SoB was 3.2 (3.8). At follow-up, 511 (53.5%) and 417 (43.7%) of the 955 participants analysed at baseline were available for analyses at the second and third assessments (T_2_ and T_3_), respectively (see Fig. 1). At follow-up, 82 participants (16.1%) and 100 participants (24.0%) had been admitted to a NH at T_2_ and T_3_, respectively.

**Table 1:** Sample characteristics at baseline (*N* = 955)

		All *N* = 955	No MCI or Dementia *N* = 295	MCI *N* = 266	Dementia *N* = 394
Socio-demographics					
Age	Mean (SD)	83.4 (5.6)	81.7 (5.6)	83.4 (5.4)	84.6 (5.5)
Females	N (%)	653 (68.4)	202 (68.5)	188 (70.7)	263 (66.8)
Married^a^	N (%)	287 (30.1)	81 (27.5)	80 (30.2)	126 (32.0)
Health condition					
GMHR^a^					
Good	N (%)	149 (15.6)	67 (22.7)	42 (15.8)	40 (10.2)
Fairly good	N (%)	378 (39.6)	127 (43.1)	109 (41.0)	142 (36.1)
Poor	N (%)	326 (34.2)	80 (27.1)	93 (35.0)	153 (38.9)
Very Poor	N (%)	101 (10.6)	21 (7.1)	22 (8.3)	58 (14.8)
P-ADL score^b^	Mean (SD)	9.1 (3.5)	7.6 (2.1)	8.4 (3.0)	10.8 (4.0)
I-ADL score^b^	Mean (SD)	5.2 (2.4)	6.6 (1.6)	5.9 (2.0)	3.6 (2.2)
CDR-SoB	Mean (SD)	3.2 (3.8)	0.8 (1.4)	1.6 (2.0)	6.1 (4.0)
Neuropsychiatric sub-syndrome score					
Agitation^c^	Mean (SD)	1.7 (4.6)	0.7 (2.7)	1.1 (3.1)	2.8 (6.1)
Psychosis^c^	Mean (SD)	0.5 (2.0)	0.1 (0.5)	0.2 (1.5)	1.1 (2.8)
Affective^c^	Mean (SD)	2.9 (5.3)	1.1 (2.6)	2.2 (3.9)	4.7 (6.8)
Use of psychotropic medication					
Antipsychotics	N (%)	36 (3.8)	5 (1.7)	7 (2.6)	24 (6.1)
Antidepressants	N (%)	148 (15.5)	32 (10.9)	38 (14.3)	78 (19.8)
Anxiolytics	N (%)	84 (8.8)	15 (5.1)	27 (10.2)	42 (10.7)
Sedatives	N (%)	211 (22.1)	54 (18.3)	59 (22.2)	98 (24.9)
Cognitive enhancers	N (%)	54 (5.7)	0	4 (1.5)	50 (12.7)
No of drugs	Mean (SD)	5.4 (2.9)	5.1 (3.1)	5.4 (2.8)	5.5 (2.9)

*MCI* Mild Cognitive Impairment, *GMHR* General Medical Health Rating Scale, *P-ADL* Personal functioning assessed with Lawton and Brody’s Physical Self-Maintenance Scale, *I-ADL* Instrumental functioning assessed with Instrumental Activities of Daily Living Scale, *CDR-SoB* Clinical Dementia Rating Scale with sum of boxes

^a^Missing information from one participant

^b^Missing information from three participants

^c^Missing information from two participants

### Prevalence, incidence and persistence of single symptoms of depression and clinically significant symptom levels

The prevalence of depressive symptoms at baseline and at follow-up is presented in Table 2. The two most prevalent single symptoms reported by the CSDD at baseline were anxiety (35.9%), and multiple physical complaints (35.2%). The prevalence at T_2_ and T_3_ were 36.3 and 33.5%, and 37.5 and 29.8% for anxiety and multiple physical complaints, respectively. At all assessments, anxiety symptoms were significantly more prevalent in participants with dementia at baseline than in those without dementia (MCI or no cognitive impairment). The prevalence of multiple physical complaints did not differ between residents with and without dementia for any assessment. The least frequent symptoms at baseline were delusion (5.6%), suicidal ideation (5.6%) and agitation (5.8%) and the prevalence of these symptoms was quite similar at T_2_ and T_3_. At baseline, 19.3% had a clinically significant symptom level indicating depression, i.e. a mean CSDD score higher than 0.42, which was equivalent to a sum-score cut-off of 9 and higher at CSDD. The cumulative incidence of a clinically significant symptom level after baseline was 15.6%. The cumulative incidence of eight items on the CSDD scale (lack of joy, irritability, retardation, lack of interest, multiple awakening, early morning awakening, poor self-esteem and delusions) was higher for those with dementia than without dementia.

**Table 2:** Prevalence, incidence and persistence of depressive symptoms (CSDD-items) by the presence of dementia at baseline

CSDD items	Prevalence					
	T_1_		T_2_		T_3_	
	All	nD / D	All	nD / D	All	nD / D
	n/N (%)	n (%) / n (%)	n/N (%)	n (%) / n (%)	n/N (%)	n (%) / n (%)
Anxiety	339/945 (35.9)	169 (30.3) / 170 (43.8)***	184/507 (36.3)	92 (30.4) / 92 (45.1)**	137/409 (33.5)	76 (28.5) / 61 (43.0)*
Sadness	273/951 (28.7)	124 (22.2) / 149 (37.9)***	134/509 (26.3)	66 (21.6) / 68 (33.5)*	99/409 (24.2)	62 (23.2) / 37 (26.1)
Lack of Joy	149/951 (15.7)	52 (9.3) / 97 (24.7)***	87/509 (17.1)	33 (10.8) / 54 (26.5)***	66/411 (16.1)	38 (14.2) / 28 (19.6)
Irritability	244/949 (25.7)	107 (19.2) / 137 (34.9)***	149/510 (29.2)	57 (18.6) / 92 (45.3)***	104/411 (25.3)	64 (23.9) / 40 (28.0)
Agitation	55/954 (5.8)	20 (3.6) / 35 (8.9)**	27/510 (5.3)	7 (2.3) / 20 (9.9)***	19/413 (4.6)	12 (4.4) / 7 (4.9)
Retardation	160/949 (16.9)	50 (8.9) / 110 (28.4)***	82/509 (16.1)	30 (9.8) / 52 (25.7)***	64/412 (15.5)	29 (10.8) / 35 (24.3)***
Multiple physical complaints	333/947 (35.2)	197 (35.2) / 136 (35.1)	190/506 (37.5)	110 (36.1) / 80 (39.8)	122/410 (29.8)	78 (29.2) / 44 (30.8)
Lack of interest	164/948 (17.3)	52 (9.3) / 112 (28.8)***	82/507 (16.2)	33 (10.8) / 49 (24.4)***	55/407 (13.5)	26 (9.8) / 29 (20.6)**
Appetite loss	215/952 (22.6)	91 (16.3) / 124 (31.6)***	88/507 (17.4)	45 (14.8) / 43 (21.2)	64/412 (15.5)	43 (16.0) / 21 (14.7)
Weight loss	146/941 (15.5)	65 (11.7) / 81 (21.0)***	53/500 (10.6)	31 (10.2) / 22 (11.2)	43/408 (10.5)	30 (11.3) / 13 (9.1)
Lack of energy	265/948 (28.0)	125 (22.3) / 140 (36.2)***	107/508 (21.1)	54 (17.6) / 53 (26.2)*	84/408 (20.6)	54 (20.2) / 30 (21.3)
Diurnal variation	85/918 (9.3)	37 (6.9) / 48 (12.7)**	72/487 (14.8)	30 (10.3) / 42 (21.3)**	44/397 (11.1)	23 (9.0) / 21 (14.8)
Difficulty falling asleep	171/922 (18.5)	101 (18.7) / 70 (18.4)	63/492 (12.8)	41 (13.8) / 22 (11.3)	62/395 (15.7)	42 (16.3) / 20 (14.5)
Multiple awakening	225/882 (25.5)	122 (23.3) / 103 (28.8)	120/478 (25.1)	58 (20.1) / 62 (32.8)**	94/386 (24.4)	60 (23.9) / 34 (25.2)
Early morning awakening	117/923 (12.7)	56 (10.2) / 61 (16.2)*	55/494 (11.1)	25 (8.4) / 30 (15.2)*	41/394 (10.4)	24 (9.5) / 17 (12.1)
Suicidal ideation	53/954 (5.6)	20 (3.6) / 33 (8.4)**	22/508 (4.3)	10 (3.3) / 12 (6.0)	16/404 (4.0)	10 (3.8) / 6 (4.3)
Poor self-esteem	128/945 (13.5)	61 (11.0) / 67 (17.3)*	80/511 (15.7)	35 (11.4) / 45 (22.1)**	55/402 (13.7)	31 (11.7) / 24 (17.4)
Pessimism	230/954 (24.1)	113 (20.2) / 117 (29.7)**	121/503 (24.1)	67 (22.0) / 54 (27.1)	73/403 (18.1)	55 (20.8) / 18 (13.0)
Delusions	53/951 (5.6)	18 (3.2) / 35 (9.0)***	22/507 (4.3)	6 (2.0) / 16 (7.9)**	19/401 (4.7)	8 (3.1) / 11 (7.9)*
Cut-off> 0.42^a^	184/955 (19.3)	68 (12.1) / 116 (29.4)***	103/511 (20.2)	47 (15.3) / 56 (27.5)**	68/417 (16.3)	42 (15.4) / 26 (17.9)
CSDD items	Incidence					
	Cumulative		T_1_-T_2_		T_2_-T_3_	
	All	nD / D	All	nD / D	All	nD / D
	n/N (%)	n (%) / n (%)	n/N (%)	n (%) / n (%)	n/N (%)	n (%) / n (%)
Anxiety	126/606 (20.8)	72 (18.6) / 54 (24.8)	83/313 (26.5)	47 (22.6) / 36 (34.3)	54/230 (23.5)	27 (16.5) / 27 (40.9)***
Sadness	121/678 (17.8)	74 (17.1) / 47 (19.3)	66/364 (18.1)	38 (16.1) / 28 (21.9)	52/267 (19.5)	34 (18.4) / 18 (22.0)
Lack of Joy	98/802 (12.2)	48 (9.5) / 50 (16.9)**	59/426 (13.8)	25 (9.1) / 34 (22.4)***	37/295 (12.5)	22 (10.9) / 15 (16.1)
Irritability	122/705 (17.3)	64 (14.2) / 58 (22.7)**	73/375 (19.5)	31 (12.5) / 42 (33.1)***	41/254 (16.1)	29 (15.8) / 12 (16.9)
Agitation	37/899 (4.1)	17 (3.1) / 20 (5.6)	21/481 (4.4)	6 (2.0) / 15 (8.2)**	11/327 (3.7)	7 (3.2) / 4 (3.7)
Retardation	93/789 (11.8)	42 (8.2) / 51 (18.3)***	52/424 (12.3)	21 (7.6) / 31 (21.2)***	37/300 (12.3)	18 (8.7) / 19 (20.2)*
Multiple physical complaints	146/614 (23.8)	93 (25.6) / 53 (21.1)	94/321 (29.3)	59 (29.6) / 35 (28.7)	42/206 (20.4)	27 (19.3) / 15 (22.7)
Lack of interest	86/784 (11.0)	42 (8.3) / 44 (15.9)**	56/415 (13.5)	26 (9.4) / 30 (21.6)**	31/296 (10.5)	16 (7.7) / 15 (17.0)*
Appetite loss	97/737 (13.2)	60 (12.8) / 37 (13.8)	51/388 (13.1)	28 (11.2) / 23 (16.8)	35/295 (11.9)	22 (11.2) / 13 (13.3)
Weight loss	81/795 (10.2)	51 (10.4) / 30 (9.8)	42/421 (10.0)	25 (9.4) / 17 (11.0)	30/304 (9.9)	20 (10.1) / 10 (9.5)
Lack of energy	113/683 (16.5)	68 (15.6) / 45 (18.2)	64/356 (18.0)	32 (13.7) / 32 (26.2)**	49/282 (17.4)	32 (16.8) / 17 (18.5)
Diurnal variation	78/833 (9.4)	39 (7.8) / 39 (11.8)	46/431 (10.7)	19 (7.2) / 27 (16.1)**	17/277 (6.1)	11 (5.8) / 6 (6.9)
Difficulty falling asleep	71/751 (9.5)	42 (9.5) / 29 (9.3)	34/387 (8.8)	20 (8.7) / 14 (8.9)	35/283 (12.4)	23 (12.4) / 12 (12.4)
Multiple awakening	119/657 (18.1)	61 (15.2) / 58 (22.7)*	68/342 (19.9)	29 (13.6) / 39 (30.2)***	44/233 (18.9)	30 (18.3) / 14 (20.3)
Early morning awakening	63/806 (7.8)	28 (5.7) / 35 (11.1)**	40/430 (9.3)	19 (7.2) / 21 (12.7)	19/287 (6.6)	8 (4.2) / 11 (11.2)*
Suicidal ideation	28/901 (3.1)	15 (2.8) / 13 (3.6)	20/482 (4.1)	9 (3.0) / 11 (5.9)	9/324 (2.8)	4 (1.9) / 5 (4.6)
Poor self-esteem	76/817 (9.3)	36 (7.3) / 40 (12.5)*	53/438 (12.1)	24 (8.8) / 29 (17.5)*	25/288 (8.7)	14 (7.0) / 11 (12.5)
Pessimism	96/724 (13.3)	62 (13.9) / 34 (12.3)	67/381 (17.6)	41 (17.0) / 26 (18.6)	24/261 (9.2)	20 (11.2) / 4 (4.8)
Delusions	29/898 (3.2)	10 (1.8) / 19 (5.4)**	14/482 (2.9)	4 (1.4) / 10 (5.3)*	13/323 (4.0)	6 (2.8) / 7 (6.4)
Cut-off> 0.42^a^	120/771 (15.6)	69 (14.0) / 51 (18.3)	68/410 (16.6)	32 (12.0) / 36 (25.2)*	35/285 (12.3)	25 (12.6) / 10 (11.5)
CSDD items	Persistence at two consecutive time points					
			T_1_-T_2_		T_2_-T_3_	
			All	nD / D	All	nD / D
			n/N (%)	n (%) / n (%)	n/N (%)	n (%) / n (%)
Anxiety			99/187 (52.9)	44 (47.8) / 55 (57.9)	62/112 (55.4)	37 (63.8) / 25 (46.3)
Sadness			66/142 (46.5)	27 (39.7) / 39 (52.7)	30/78 (38.5)	18 (45.0) / 12 (31.6)
Lack of Joy			28/82 (34.1)	8 (25.8) / 20 (39.2)	14/49 (28.6)	6 (28.6) / 8 (28.6)
Irritability			75/133 (56.4)	25 (43.9) / 50 (65.8)*	43/91 (47.3)	23 (54.8) / 20 (40.8)
Agitation			6/29 (20.7)	1 (11.1) / 5 (25.0)	4/19 (21.1)	1 (16.7) / 3 (23.1)
Retardation			9/28 (32.1)	2 (22.2) / 7 (36.8)	14/46 (30.4)	6 (31.6) / 8 (29.6)
Multiple physical complaints			92/180 (51.1)	51 (48.1) / 41 (55.4)	55/139 (39.6)	34 (40.5) / 21 (38.2)
Lack of interest			26/87 (29.9)	7 (24.1) / 19 (32.8)	13/46 (28.3)	5 (33.3) / 8 (25.8)
Appetite loss			36/117 (30.8)	16 (30.8) / 20 (30.8)	13/50 (26.0)	9 (33.3) / 4 (17.4)
Weight loss			9/71 (12.7)	5 (15.2) / 4 (10.5)	4/34 (11.8)	4 (19.0) / 0
Lack of energy			42/148 (28.4)	22 (30.6) / 20 (26.3)	19/62 (30.6)	12 (35.3) / 7 (25.0)
Diurnal variation			22/43 (51.2)	10 (47.6) / 12 (54.5)	13/44 (29.5)	5 (33.3) / 8 (27.6)
Difficulty falling asleep			26/91 (28.6)	19 (32.8) / 7 (21.2)	13/42 (31.0)	11 (39.3) / 2 (14.3)
Multiple awakening			39/104 (37.5)	22 (38.6) / 17 (36.2)	22/74 (29.7)	14 (36.8) / 8 (22.2)
Early morning awakening			14/56 (25.0)	6 (23.1) / 8 (26.7)	9/39 (23.1)	7 (35.0) / 2 (10.5)
Suicidal ideation			2/26 (7.7)	1 (9.1) / 1 (6.7)	3/16 (18.8)	2 (33.3) / 1 (10.0)
Poor self-esteem			26/69 (37.7)	11 (32.4) / 15 (42.9)	20/52 (38.5)	12 (50.0) / 8 (28.6)
Pessimism			54/121 (44.6)	26 (41.9) / 28 (47.5)	35/76 (46.1)	25 (58.1) / 10 (30.3)*
Delusions			8/23 (34.8)	2 (22.2) / 6 (42.9)	2/14 (14.3)	1 (20.0) / 1 (11.1)
Cut-off> 0.42^a^			35/101 (34.7)	15 (37.2) / 20 (32.8)	16/65 (24.6)	8 (26.7) / 8 (22.9)

*** *p* < 0.001; ** *p* < 0.01; * *p* < 0.05 (results of generalized linear mixed model adjusting for within-municipality correlations; SAS GLIMMIX procedure)

*CSDD* Cornell Scale for Depression in Dementia, *nD* no Dementia, *D* Dementia

^a^A mean CSDD score higher than 0.42 was defined as clinically significant symptom level indicating depression, and is equivalent to a sum-score cut-off of 9 and higher for CSDD

The persistence of clinically significant levels of depressive symptoms between T_1_ and T_2_ was 34.7%, while the persistence between T_2_ and T_3_ was 24.6%. Only one depressive symptom, irritability, was more persistent between T_1_ and T_2_ for those with dementia at baseline (65.8%) than for those without (43.9%). The symptom pessimism was more persistent between T_2_ and T_3_ in those without dementia at baseline compared to those with dementia, i.e. 58.1 and 30.3%, respectively.

### Mean depressive symptoms score over the follow-up period

**Fig. 2:**
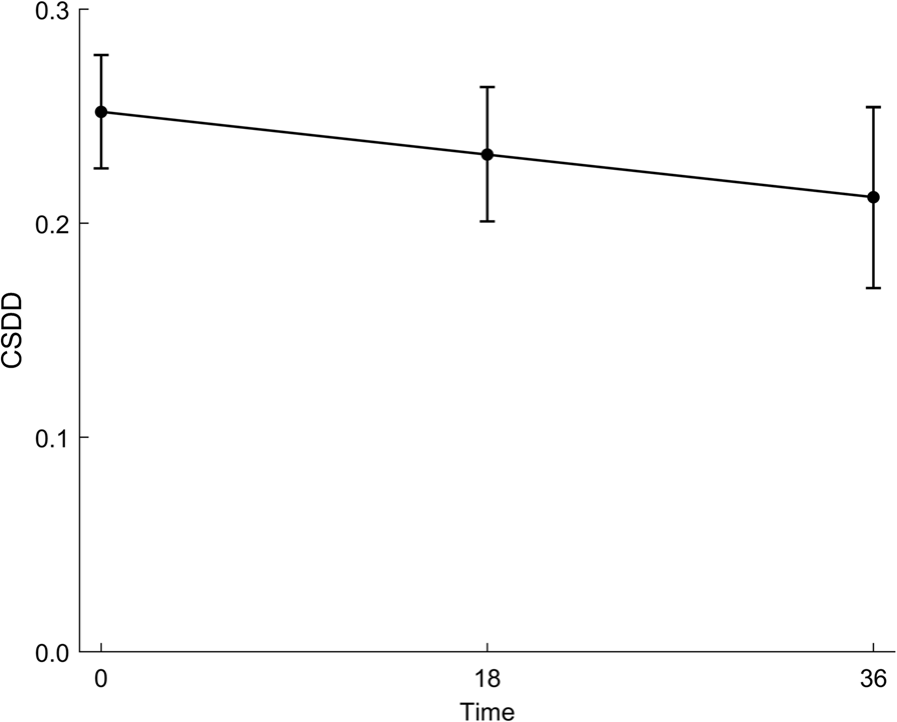
Time trend in mean CSDD, unadjusted

The overall mean CSDD score decreased over the 36-month follow-up (Table 3 and Fig. 2). Unadjusted for covariates, there was a significant linear reduction in mean CSDD score through the study period (*p* = 0.015), by an average of 0.001 points per month.

**Table 3:** CSDD mean score at each time point in total and stratified by dementia status at baseline

	T_1_		T_2_		T_3_	
	All	D/nD	All	D/nD	All	D/nD
CSD						
N	955	394/561	511	204/307	417	145/272
Mean (SD)	0.25 (0.29)	0.34 (0.34) / 0.19 (0.23)	0.24 (0.26)	0.32 (0.30) / 0.18 (0.22)	0.21 (0.26)	0.24 (0.27) / 0.19 (0.26)

*CSDD* Cornell Scale for Depression in Dementia, *nD* no Dementia, *D* Dementia

### Association between dementia and mean depressive symptom score

**Fig. 3:**
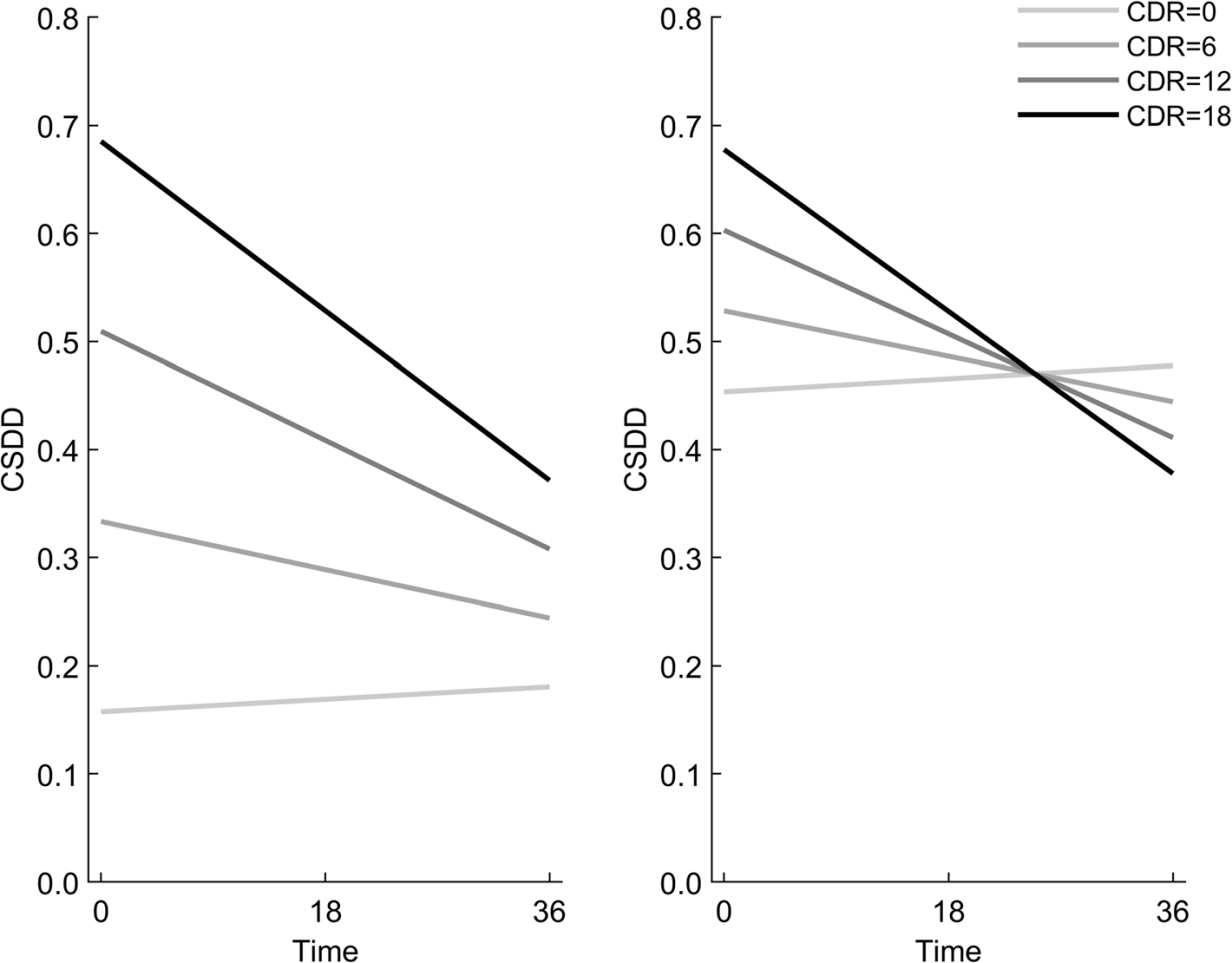
Interpretation of Model 1, unadjusted (left) and adjusted (right). CDR: CDR-SoB = Clinical Dementia Rating Scale with sum of boxes

The association between the severity of cognitive decline, assessed with the CDR-SoB and the mean CSDD score over time was studied in two multiple models. In multiple Model 1 (see Table 4), the association between CDR-SoB at T_1_ and mean CSDD score changed over time (*p* < 0.001 for interaction). More severe cognitive impairment was associated with a higher mean CSDD score at T_1_, with the association significant for values of CDR-SoB of 3 or higher and becoming somewhat stronger with increasing values of CDR-SoB (*p* < 0.001). However, this association was not significant at T_2_ and at T_3_ (Fig. 3).

**Table 4:** Associations between CDR-SoB measured at baseline (Model 1) or longitudinally (Model 2) and CSDD mean score^a^

Covariates	Model 1				Model 2			
	Unadjusted models		Adjusted AIC-reduced model^b^		Unadjusted models		Adjusted AIC-reduced model^c^	
	Regr.coeff. (SE)	*p*-value	Regr.coeff. (SE)	*p*-value	Regr.coeff. (SE)	*p*-value	Regr.coeff. (SE)	*p*-value
Main variables								
Time	0.0006 (0.0006)	0.244	0.0007 (0.0005)	0.191	−0.0002 (0.0005)	0.690	−0.0003 (0.0004)	0.530
CDR-SoB	0.03 (0.002)	**< 0.001**	0.01 (0.003)	**< 0.001**	0.03 (0.002)	**< 0.001**	0.01 (0.002)	**< 0.001**
CDR-SoB*Time	−0.0005 (0.0001)	**< 0.001**	−0.0005 (0.0001)	**< 0.001**	− 0.0005 (0.00009)	**< 0.001**	−0.0003 (0.00008)	**< 0.001**
Socio-demographic information								
Women	0.04 (0.01)	**0.006**	0.03 (0.01)	**0.027**	0.04 (0.01)	**0.002**	0.03 (0.01)	**0.009**
Age (years)	−0.004 (0.001)	**< 0.001**	−0.004 (0.001)	**0.001**	−0.005 (0.001)	**< 0.001**	− 0.002 (0.0009)	**0.026**
Single	0.009 (0.01)	0.515			0.01 (0.01)	0.330		
General physical health								
GMHR								
Very poor	0.08 (0.03)	**0.003**			0.08 (0.03)	**0.002**	0.07 (0.02)	**0.001**
Poor	0.02 (0.02)	0.393			0.04 (0.02)	0.067	0.03 (0.02)	0.054
Fair	0.008 (0.02)	0.635			0.0008 (0.02)	0.968	0.004 (0.02)	0.813
Good – ref.	0							
P-ADL	0.0006 (0.002)	0.791			−0.001 (0.002)	0.577		
I-ADL	−0.003 (0.004)	0.501	−0.008 (0.004)	**0.047**	0.0005 (0.004)	0.899		
Neuropsychiatric sub-syndrome score								
Agitation	0.02 (0.001)	**< 0.001**	0.008 (0.001)	**< 0.001**	0.02 (0.001)	**< 0.001**	0.01 (0.001)	**< 0.001**
Psychosis	0.02 (0.003)	**< 0.001**			0.02 (0.003)	**< 0.001**		
Affective	0.02 (0.001)	**< 0.001**	0.02 (0.001)	**< 0.001**	0.03 (0.001)	**< 0.001**	0.02 (0.001)	**< 0.001**
Use of psychotropic medication								
Antipsychotics	0.004 (0.03)	0.891			−0.02 (0.03)	0.476		
Antidepressants	0.03 (0.02)	0.086			0.04 (0.02)	**0.032**		
Anxiolytics	0.009 (0.02)	0.674			0.03 (0.02)	0.113		
Sedatives	0.03 (0.01)	**0.024**	0.03 (0.01)	**0.030**	0.04 (0.01)	**0.006**		
Cognitive enhancers	0.05 (0.03)	0.079			0.01 (0.03)	0.588		
NH resident beforeT_3_					−0.12 (0.03)	**< 0.001**	−0.07 (0.02)	**0.001**

*AIC* Akaike’s Information Criterion, *CDR-SoB* Clinical Dementia Rating Scale with sum of boxes, *CSDD* Cornell Scale for Depression in Dementia, *GMHR* General Medical Health Rating Scale, *P-ADL* Personal functioning assessed with Lawton and Brody’s Physical Self-Maintenance Scale, *I-ADL* Instrumental functioning assessed with Instrumental Activities of Daily Living Scale, *NH* Nursing home. Bold *p*-values are significant

^a^Results of linear mixed model

After exclusion of cases with at least one missing value on covariates, in Model 1: *N* = 946 at T_1_, *N* = 505 at T_2_ and *N* = 412 at T_3_ and in Model-2: *N* = 946 at T_1_, *N* = 497 at T_2_ and *N* = 403 at T_3_. Random slope for time included

^b^Adjusted for covariates measured at baseline

^c^Adjusted for covariates measured simultaneously except for gender, age and living alone

**Fig. 4:**
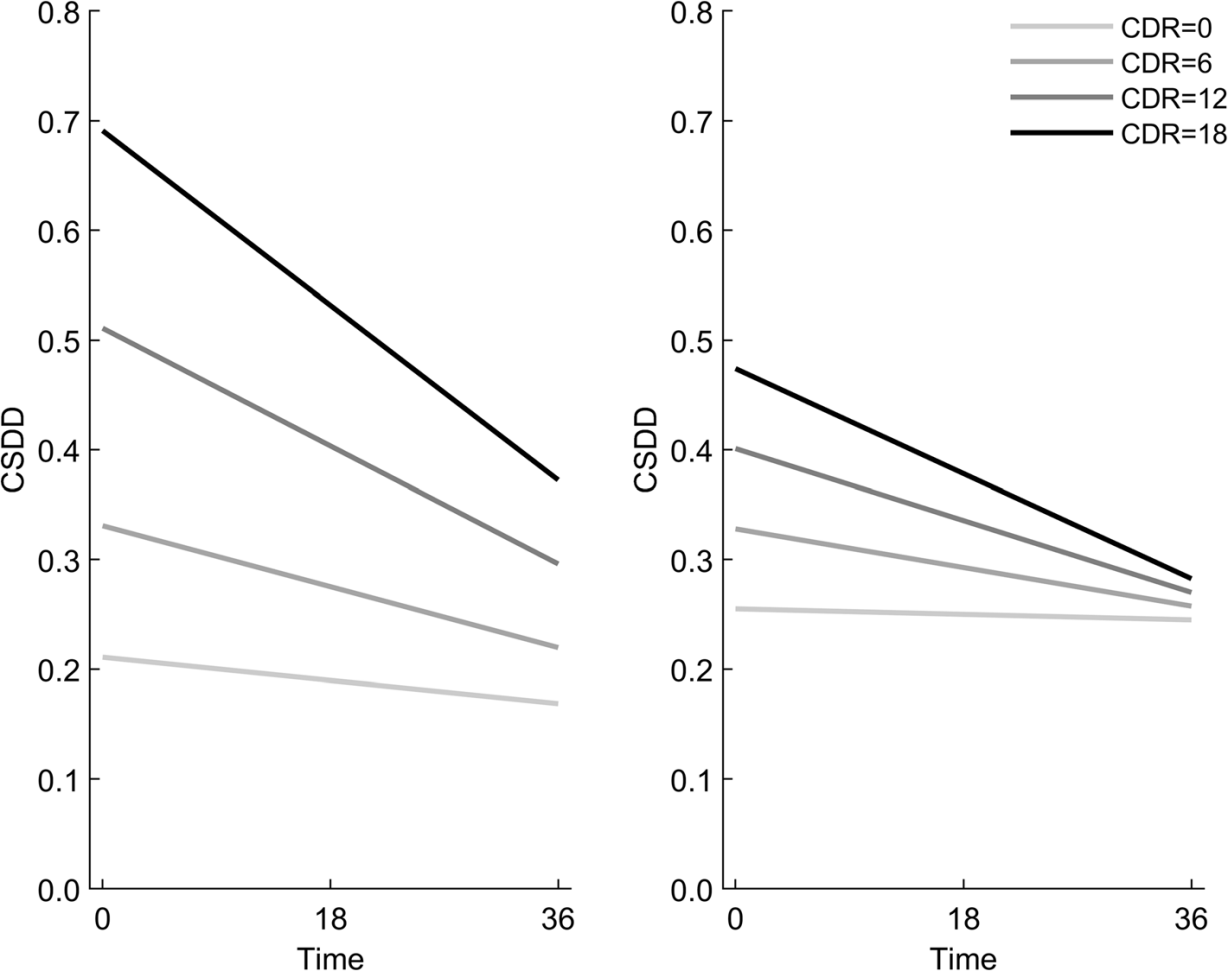
Interpretation of Model 2, unadjusted (left) and adjusted (right). CDR: CDR-SoB = Clinical Dementia Rating Scale with sum of boxes

In multiple Model 2, the association between CDR-SoB and mean CSDD score assessed simultaneously at three time points also changed over time (*p* < 0.001 for interaction). The association was positive and significant for values of CDR-SoB of 2 or higher, and became somewhat stronger with increasing values of CDR-SoB at both T_1_ and T_2_ (*p* < 0.001), but was not significant at T_3_ (Fig. 4).

In both multiple models, being female, younger, and having a higher level of agitation and affective sub-syndrome score were associated with a higher score on the CSDD scale. In addition, in multiple Model 1, using sedatives and having a lower I-ADL score at baseline were associated with higher score on the CSDD scale throughout the follow-up. In Model 2, having very poor, as compared to good, physical health and not being a NH resident at T_2_ and T_3_ were associated with a higher score on the CSDD scale when measured simultaneously.

## Discussion

The present study describing the prevalence, incidence and persistence of depressive symptoms over a 36-month follow-up period among older adults receiving in-home care at baseline, and found that both the baseline prevalence and cumulative incidence of depressive symptoms were higher in those with dementia at baseline than in those without dementia at baseline. However, the persistence of single depressive symptoms did not differ between those with or without dementia at baseline. When exploring the association between cognitive impairment and the course of depressive symptoms over time, we found a positive association between the severity of cognitive impairment and mean depressive symptoms assessed simultaneously. Furthermore, we found that the association changed over time and was not significant at the last follow-up, 36 months after baseline. In the same analysis, being younger, female, in very poor physical health, with more neuropsychiatric symptoms and not being a NH resident were also associated with higher mean depressive symptoms. This is the first study exploring depressive symptoms over time in older people receiving in-home care at inclusion.

When looking at single symptoms assessed with CSDD, most symptoms at baseline were more common in participants with dementia than in participants without dementia. However, the differences in prevalence of single symptoms among those with and without dementia at baseline were reduced over the 36-month follow-up. This might be explained by an increased cognitive decline both in those with and without baseline dementia, which would lead to a more homogenous group of participants. Among those without dementia at baseline, almost half had mild cognitive impairment. The turnover rate from mild cognitive impairment to dementia is high within the following year in older adults. However, not all with mild cognitive impairment will get dementia [49]. The persistence of symptoms at two consecutive assessments did not differ between those with and without dementia at baseline. Even so, the cumulative incidence of several symptoms was higher in those with dementia at baseline. Most of the symptoms with a higher cumulative incidence in participants with dementia (i.e. lack of joy, irritability, retardation, lack of interest, multiple awakening, early morning awakening) are classified as non-mood factors in CSDD [50]. Of these non-mood symptoms, irritability was a symptom with both a higher cumulative incidence and baseline prevalence in those with dementia at baseline. Other studies exploring symptoms of depression in older adults have found irritability to be highly prevalent in community-dwelling residents with dementia [51], in NH residents where dementia is very prevalent [52] and in outpatients with dementia and depression [53]. It can be difficult to determine whether irritability is a symptom of depression or dementia [52]. Sleep disturbances, including multiple awakening and early morning awakening, were among the few symptoms in CSDD where the baseline prevalence did not differ between those with and without dementia, but those with dementia at baseline had a higher cumulative incidence throughout the follow-up period. Sleep disturbances can be explained by several medical conditions in older adults with and without dementia. However, it is suggested that these symptoms should be considered to be unspecific symptoms of depression in geriatric populations [54, 55]. Poor self-esteem and delusion are symptoms included in the CSDD mood factor [50] that had a higher cumulative incidence in those with dementia at baseline than those without. It might be that some symptoms both in the non-mood and mood factors of CSDD have a higher variability in those with baseline dementia than without. Even so, single symptoms were not adjusted for deaths or loss of follow-up, cognitive decline or development of dementia during the follow-up period. Additionally, the time between the assessments was more than 1 year.

The prevalence of clinically significant depressive symptoms in the present study of older adults with in-home care needs was 19% at baseline, which is higher than the diagnostic pooled prevalence of depression in older adults [8] and the prevalence reported in general community-dwelling living residents in Hong Kong [56], but lower than in an American study of among older people receiving in-home care (24.1% with depression) [57]. Those with dementia were more likely to have clinically significant depressive symptoms than those without dementia at baseline, which was expected since depression is associated with dementia [58]. The prevalence was comparable to the prevalence found with use of CSDD in older adults with dementia receiving in-home care in other Nordic countries such as Sweden and Finland (30%) [10], but lower than the prevalence the prevalence found in other European countries, including Estonia and Germany (49 and 60%, respectively) [10]. The care needs, the severity of cognitive decline and prevalence of physical comorbidity in these samples are unknown. However, we may speculate that due to the political and cultural similarities in the Nordic countries, both the type of formal care delivered and the dementia characteristics and physical comorbidity of older people receiving in-home care are comparable, and thus, it is reassuring that the prevalence of depression was similar.

In the present study, there was a significant but small decline in mean depressive symptom score assessed with CSDD from baseline to the last follow-up. Few longitudinal studies have explored depression in older adults receiving in-home care, but a decline in depressive symptom score over time has also been found in previous studies from the geriatric psychiatry service [56], and NH setting [52]. As those studies have pointed out, we do not know if the results are a part of the natural course of depression when dementia is prevalent, or are just artefacts [52, 56]. It could be the Hawthorne effect, namely that being involved in a research context influences the behaviour of the respondents’ caregiver and/or research assistants who conduct the interviews. Thus, some participants may be scored with fewer or less severe symptoms over time than they actually have. Even so, the decline in the mean score of depressive symptoms per month was 0.001 and of less clinical importance.

In the adjusted analysis where both cognitive impairment and depressive symptoms were assessed simultaneously (Model 2), more severe cognitive decline was associated with more severe depressive symptoms. The association was stronger with more severe cognitive decline, but only at T_1_ and T_2_. This study reports an association between the severity of cognitive decline and the severity of depressive symptoms, and it is reasonable to believe that the underlying neuropathological condition causing cognitive decline, MCI and dementia can also cause depressive symptoms [9]. Shared biological pathways between depression and dementia such as vascular disease, increased deposition of beta amyloid plaques and neuroinflammation have been suggested [59]. However, the association between cognitive decline and the severity of depressive symptoms was stronger at baseline than at T_2_, and there was no association at T_3_. It may be that, as suggested by others, individuals with the most severe cognitive decline and who are at a later stage of dementia at the last assessment no longer have the cognitive capacity necessary to communicate depressive symptoms [52]. In addition, at the last assessment, only 40% of the original sample was able to participate, and we may speculate that those surviving the follow-up period were less likely to have or express depressive symptoms, even after adjusting for age [52]. Higher age was associated with less severe depressive symptoms in our analytic model. Others have explained this as older adults being “survivors” who are less prone to becoming depressed [60] or show depressive symptoms [52].

At T_2_ and T_3_, 82 and 100 participants were NH residents, respectively. In the adjusted analysis, NH residents had lower depressive scores compared to those receiving in-home care. In line with our results, a European study that included data from patients from eight countries found that the prevalence of depression was higher in older adults with dementia receiving in-home care than in those admitted to NH [10]. We do not know the reason for our findings. However, a well-known reason for NH admission, in addition to poor cognition, impaired activities of daily living and comorbidity, is mental health problems, such as depression and other neuropsychiatric symptoms [61]. Thus, it is likely that treatment, care or the setting is important for depressive symptoms. Lower depressive symptom scores in NH residents may be due to more social interaction in NHs than among those living at home. Individuals in NH may have received treatment for their depressive symptoms. Thus, NH residents may not have depressive symptoms to the same extent as those receiving in-home care. Moreover, it could be that the primary caregiver for those living at home rated depressive symptomatology high due to caregiver distress. Another possibility is that primary caregivers in NHs do not know their residents well enough to understand how residents express their mental states, or this expression is overlooked, which would be less likely for the primary caregiver for participants who live at home. This would make the mean scores lower in NH residents. This needs further exploration.

Poor physical health is a well-known risk factor for depression [58], and thus, we adjusted for physical health in our analysis. We found that higher agitation and affective sub-syndrome score at each time point were independently associated with higher depressive symptom scores assessed simultaneously. This was expected since the CSDD includes items that also may represent neuropsychiatric symptoms.

The study has a number of strengths, such as using a well-known, internationally recognized scale for assessing depressive symptoms [62], a high number of baseline participants, use of a direct measure of cognitive functioning, and controlling for several variables of potential importance to the outcome, such as activity of daily living, physical health, and demographic variables. Even so, it has some limitations that need to be described.

First, the study findings should be interpreted with caution since this is a study about associations and should not be mixed with causality.

Secondly, our participants were younger and were more often male than individuals who were not included [27]. Those who declined to participate may not be at random. Thus, the representativeness of the sample for older people receiving in-home care in Norway may be somewhat hampered. Furthermore, the sample is not representative for general older people living in a community, since one inclusion criterion was that the participant received in-home care. Thus, caution should be taken in generalizing the study results.

Thirdly, it is challenging to conduct a longitudinal study of depressive symptoms in older people with in-home care needs over a three-year-period. The time between the assessments was 18 months and should have been shorter to give a better understanding of incidence and persistence of single symptoms from the CSDD among those with and without dementia. Furthermore, repeated measures of the same individuals contributes to dependency in the data. Moreover, the large number who left the study mainly due to mortality, gives unequal number of observations per participant, and this, generates imbalance in the data. In the present study we used linear mixed model, since this statistics is known to handle imbalanced data of any degree by including all available data [63]. Furthermore, this statistics allows time-varying covariates to be included in the model and it review correlations among repeated measurements well [63].

## Conclusion

In the present study, the baseline prevalence and the cumulative incidence of depressive symptoms were higher in participants with dementia at baseline than in those without. The severity of cognitive decline and mean depressive symptoms was positively associated, but the association became weaker over time. Our study shows that depression and dementia are interconnected, and that nurses and clinicians should pay attention to cognitive status when observing or evaluating depression among older adults receiving in-home care. When dementia is suspected, the individual must be offered a diagnostic work-up in order to be given the best treatment possible, which also is in line with the national policy documents [64].

## Data Availability

The data belong to the Research Centre for Age-related Functional Decline and Disease, Innlandet Hospital Trust, and will not be shared due to The Regional Committee for Medical and Health Research Ethics and Norwegian regulations.

## Abbreviations

AIC: Akaike’s Information Criterion
ATC: Anatomical Therapeutic Chemical Classification System
CDR-SoB: Clinical Dementia Rating Scale with sum of boxes
CI: Confidence Interval
CSDD: Cornell Scale for Depression in Dementia
D: Dementia
GMHR: General Medical Health Rating Scale
I-ADL: Instrumental functioning assessed with Instrumental Activities of Daily Living Scale
MCI: Mild Cognitive Impairment
N: Number
nD: No Dementia
NH: Nursing home
NPI: Neuropsychiatric Inventory
P-ADL: Personal functioning assessed with Lawton and Brody’s Physical Self-Maintenance Scale
SD: Standard Deviation
SE: Standard Error
